# Sex differences in implicit processing of allocentric relationships between objects and location in a Simon task

**DOI:** 10.1371/journal.pone.0235964

**Published:** 2020-07-22

**Authors:** Matthew Mosso, Adam Freudenberg, Kristofer McCracken, Robert F. McGivern

**Affiliations:** Department of Psychology, San Diego State University, San Diego, CA, United States of America; University of Pittsburgh, UNITED STATES

## Abstract

Simon tasks reveal implicit processing conflicts that arise when the abstract coding of stimulus position is incongruent with coding for location of the output response. Participants were tested with two versions of a Simon task in a counterbalanced order to examine a potential female bias for attending to object characteristics versus object location. Both tasks used a triangle pointing to the left or right. A simple version presented the triangle in an inner or outer position relative to central fixation. A more complex version included a frame surrounding the inner-outer triangle presentation area in order to introduce additional visual elements for left/right visual processing. When the No Frame version was the first presented, there were no sex differences in the Simon effect in either version, which is consistent with results from other studies that did not provide feedback regarding accuracy. When the initial test was the Frame version, we observed a reverse Simon effect for incongruent triangles presented in the left inner position, with females faster than males to identify the incongruent condition versus the congruent (-59 vs -5 msec). In the No Frame condition that followed, females showed a carryover effect from the previous Frame condition, exhibiting positive Simon effects that were two fold larger than males for identifying incongruent stimuli presented in the left and right outer positions. Similar to previous Simon studies, females showed longer overall reaction times than males (~15%). The difference was not related to the Simon effect and is also found in other types of tasks involving early visual processing of objects with location. Based on sex differences in the Simon effect that emerged following initial experience of the triangle adjoining the frame, the present results support a female bias toward broader integration of objects within the context of location.

## Introduction

Simon tasks demonstrate the role of early sensory processing for aligning the spatial representation of a stimulus with the egocentric coding of the output response [1]. It was first demonstrated in an auditory task, where a command of ‘left’ or ‘right’ was presented to the left or right ear of the participant, who was then required to press a key with the hand that corresponded to the command [2]. Presenting the command to the anatomically congruent ear (e.g., ‘left’ to the left ear) led to a faster reaction time compared to when it was presented to the incongruent ear. This difference in responding to incongruent versus congruent stimuli is called the Simon effect.

Subsequent studies revealed that the effect arises from a conceptual conflict between the abstract spatial code for the stimulus, as it exists within the perceptual environment, and the egocentric code related to the spatial mapping of the response output. It is not restricted to the anatomical relationships implied by a left or right-handed response. The Simon effect also occurs when participants respond using different fingers on the same hand, as well as crossing their hands to discriminate left-right or up-down relationships between objects presented to the same side of the body [3–7]. The importance of bottom-up processing in producing the Simon effect is indicated by the fact that the Simon effect dissipates as the interval between stimulus and response lengthens to allow top-down analysis [1].

Simon’s early studies tested equal number of males and females and found that reaction time in females was consistently longer than males for identifying both congruent and incongruent stimuli. Interestingly, the sex difference in reaction time was not associated with any differences in the Simon effect or accuracy [2,8,9]. Perhaps for this reason, sex was rarely included as a variable in Simon task studies conducted in the succeeding decades. More recently however, two studies have observed a larger Simon effect in females compared with males [10,11]. Both studies also observed a longer reaction time in females, but the difference did not account for the sex difference in the Simon effect. One study also observed better accuracy in males for recognizing congruent stimuli compared to females [11].

Longer reaction times suggest greater implicit processing of spatial relationships among objects proximal to the stimulus in females compared to males. Support for this possibility comes from several studies. First, females performing a Simon task show greater neural activation of the temporal cortex than males in spite of the absence of any sex difference in the Simon effect [12]. Second, female reaction times are 10–15% longer than males in other types of studies that directly manipulate the perception of simple visual elements related to location and object characteristics [13,14]. For instance, longer female reaction times were found in a match to sample experiment, where a red or blue circle appeared in different locations within a square frame. Based on accuracy, females also exhibited an implicit bias for detection of color versus location compared to males. Males exhibited a bias toward attending to location. However, these sex differences emerged only when the order of presentation was analyzed and were not related to sex differences in reaction time. Females were more accurate than males in discriminating color when color was tested before location, while males were more accurate for discriminating location when it was the first tested. These sex differences were greatly attenuated or absent when an endogenous cue was added that predicted the right or left location for the appearance of the matching stimulus, indicating that the sex difference arises from bottom-up processing that can be overridden by further top-down analysis [14].

Early visual processing that integrates retinotopic coding of form, motion, and color provides a functional basis for downstream determinations of ‘where’ a stimulus is in the environment and ‘what’ it is [15,16]. This hierarchical analysis of sensory information occurs in parallel within the dorsal and ventral streams that extend from the primary visual cortex into the parietal and inferior temporal cortex, respectively. While the streams are anatomically and functionally distinct, interconnections at multiple levels of processing ensure each has information about processing within the other [17,18].

Although early visual processing functionally conjoins object and location, the context and details needed for perception arise from downstream interactions [19–20]. Each visual stream has reciprocal connections with distinct areas of the prefrontal cortex. These connections are integrated into attentional networks that can feedback on early sensory processing to accommodate cognitive goals. The dorsal attention network (DAN), which has its hub in the dorsolateral prefrontal cortex, is reciprocally connected to dorsal areas in the posterior parietal lobe and the frontal eye fields. It plays an essential role in top down processing for selective and covert attention. The ventral attentional network (VAN) is a bottom-up attentional system involving connections between the ventral prefrontal cortex and the temporo-parietal junction. It plays a primary role in orienting to novel or unexpected stimuli and is strongly lateralized to the right hemisphere [21].

Sex differences are found in visual processing within these attentional networks, as well as in sub processes that relate to orienting, selective attention and working memory. Dumais et al. [22] observed greater DAN greater activation in adult females in response to reward/punishment cues. In a study of visuo-spatial attention allocation in individuals between 13 and 38 years of age, Rubia et al. [23] found greater female activation in right-hemispheric inferior frontal and superior temporal regions compared to greater activation in males in left-hemispheric inferior temporo-parietal regions. The pattern emerged with age, suggesting the sex differences in visuo-spatial attention arose from asynchronous maturation of these brain regions.

Lower-level processes serving these attentional networks also show sex differences. Liu et al. [24] observed faster orienting responses in females to covertly attend to a spatially cued location. Selective attention studies show that females are more influenced than males in using a valid endogenous cue to predict a stimulus location. Bayliss et al. [25] employed a Posner type of cuing task to study selective attention using two types of endogenous cues, a frontal face with eyes that looked to the right or left, and a horizontal arrow cue that pointed left or right. A female advantage was found for responding to valid facial cues, but only when the stimulus onset asynchrony (SOA) was relatively long (700 msec). Females also showed an advantage over males for responding to the endogenous arrow cue at SOAs of 300 and 700 msec. Interestingly, female reaction time was longer than males in responding to the arrow cue, but not the facial cues. When the authors employed exogenous cues, no sex differences were found in reaction time or validity responses. Merrit et al. [26] also found longer reaction times in females, as well as greater validity effects compared to males when they used an endogenous arrow cue, but no sex differences when they used an exogenous cue. Finally, Stoet [27] studied selective attention using a flanker task enhanced with a g/no-go procedure that provided online feedback about response accuracy. Results showed a greater disruptive influence of flankers on female accuracy compared to males, as well as longer female reaction times to respond to invalid flankers. The pattern indicates greater female attention to proximal information in the stimulus environment.

Working memory systems also show sex differences in connectivity that correspond to behavioral studies. In females, working memory networks are more strongly connected to limbic structures and right prefrontal areas than males. In contrast, males show greater connectivity to posterior parietal areas. This pattern is consistent with observed sex differences in object working memory versus visuo-spatial working memory [28,29]. Spatial working memory involves connections between the dorsolateral prefrontal cortex and the posterior parietal cortex, whereas object information in working memory involves connections between the ventrolateral prefrontal cortex and the temporal lobe [30]. The two systems are integrated with perception according to task demands. For instance, visuo-spatial and object working memory are coordinated to facilitate attentional allocation for object recognition within the ventral stream [31]. If a sex difference occurs in the relative integration of the information from the two systems, it would be expected to manifest as a sex-related bias in the perception of a stimulus relative to its location versus its physical characteristics.

Simon tasks inherently require integration of object characteristics with spatial location, which involves processing of egocentric and allocentric relationships among the stimuli in the environment, as well as the location of the output response. Allocentric and egocentric perspectives involve distinct regions of the superior and medial aspects of the dorsal stream respectively, each of which can support visual priming [32, 33]. Even when a Simon task contains irrelevant visual information for target discrimination, neural processing of both explicit and implicit relationships are detectable in the averaged evoked response and can influence performance [34]. However, the principle of visual proximity appears to be essential for this effect. If independent elements in the visual field are not implicitly joined by proximity or task requirements, the Simon effect disappears because of the additional processing required for conscious visual assessment [6,35].

The direction of the Simon effect is usually positive, meaning that there is an increase in reaction time to identify an incongruent stimulus versus one that is congruent. However, a reverse Simon effect has been observed in an auditory paradigm where participants were presented with the instruction of ‘left’ or ‘right’ in one ear and asked to respond with the hand designated by the command [9]. A command of ‘left’ given to the right ear showed the expected positive Simon effect. However, when participants were presented with a distracting noise simultaneously in the ‘right’ ear, females seemed to incorporate this task irrelevant information to help them recognize incongruent significantly faster than congruent stimuli. This did not occur in males. This facilitating effect produced by an irrelevant stimulus is consistent with the hypothesis that females implicitly process allocentric spatial relationships in the stimulus environment in more depth than males [13,14].

Because the Simon task is known to involve early processing of object/location integration we hypothesized that when the visual environment in a Simon task included non-essential allocentric relationships with another stimulus, females would be more affected than males and show larger Simon effects. Two versions of a Simon task were created and administered to all participants in a counterbalanced order. The simplest version used an equilateral triangle that pointed to the left or right. On a given trial, the triangle was presented in an inner position or outer position relative to fixation. The more complex version used the same stimuli, but with a box frame surrounding the presentation area for the triangle. The purpose was to create an additional left/right allocentric relationship between the target stimulus and its proximal environment. In the simple version without the box frame, we expected to replicate Simon’s early studies showing no sex difference in the Simon effect. In the more visually complex frame version, we expected females to show a larger Simon effect than males if the added non-essential information interfered with determining congruency, but a reduced or reverse Simon effect if the added information enhanced the assessment of congruency. We now report sex differences that were partly confirmed, but emerged in a more complex manner than anticipated.

## Materials and methods

### Participants

54 females (age = 19.1 ± 0.67, SD) and 39 males (age = 19.4 ± .89, SD), all right handed, were recruited from the undergraduate population of San Diego State University. Participants were informed that the experiment was designed to assess speed for detecting whether a stimulus pointed to the left or right side of the computer screen. All participants provided written informed consent and received compensation. Procedures were approved by the Committee on Protection of Human Subjects.

### Apparatus and stimuli

Stimulus presentation software was developed by Eugene Terehov (https://www.linkedin.com/in/eterehov) and the program was run on an Apple iMac with a 24” screen. Stimuli were created in Adobe Illustrator (version CS4) and saved in a Portable Network Graphics format. The application and the stimuli employed are available at no cost for non-commercial research purposes by contacting the corresponding author.

The task employed an equilateral triangle that pointed toward the left or right side of the computer screen. Participants were instructed to respond to a triangle that pointed to the left side of the screen by pressing the ‘q’ key with their left hand and to a right pointing triangle by pressing the ‘p’ key with their right hand. This created stimulus–response trials that were congruent (i.e., when a right pointing triangle was on the right side of the screen, which required a right hand response) and incongruent (i.e., when a left pointing stimulus was on the right side of the screen, which required a left hand response).

The computer screen was centered at eye level at a distance of approximately 50 centimeters. Stimuli were presented in one of two locations on each side of a plus sign that served as a central fixation point. The screen presentation area for the task was 19.5cm x 19.5cm, with a white background. The fixation point was a plus sign comprised of black lines that were 14mm in length with a thickness of 0.75mm. The equilateral triangle used as a stimulus was dark gray in color (RGB settings: 133, 130,128) and 18mm^2^ in area. The triangle was employed instead of an arrow because it contained less salient semantic information related to direction.

At the beginning of each version, participants were presented with 20 practice trials used to familiarize them with the response keys, as well as to obtain simple RT for left and right responses. On these trials, a 15 mm diameter black circle was shown for 400milliseconds (ms) to the right or left of center at a visual angle of 6° for 20 trials. If the circle appeared on the left, they were instructed to press the q key; if on the right they were to press the p key. All trials began with the appearance of the plus sign for 400ms, followed by a variable interval (350–750ms) before the stimulus appeared for 400ms. The next trial began 500ms after the participant’s response. The plus sign remained on the screen between trials and disappeared only when a pop-up screen appeared after each set of 20 trials to indicate that the participant could take a break. Trails were resumed once the participant pressed the space bar.

Following the practice trials, participants were told they would see a triangle appear on the right or left part of the screen and use the keys to indicate if the triangle pointed to the right or left side of the computer screen. In the first version (No Box), the triangle appeared to the right or left of the fixation point in one of the two horizontal positions on each side. The visual angle of the outer position was 8° and the inner position 5°. The eighty experimental trials were presented in a pseudorandom order that varied by position relative to the plus sign (inner vs outer), arrow direction (pointing left vs right) and side of presentation (left or right).

**Fig 1:**
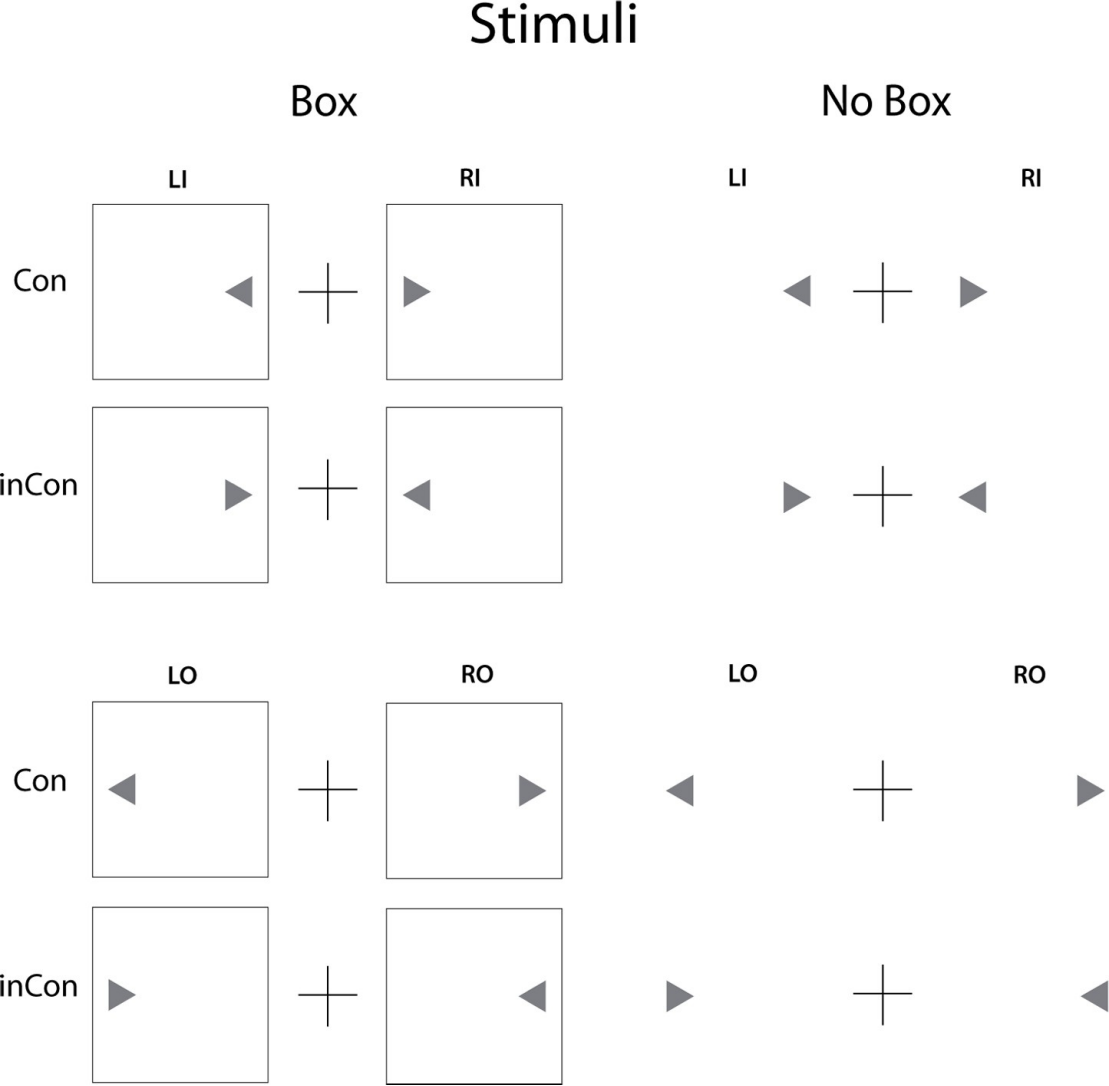
Shown are the congruent (Con) and incongruent (inCon) stimuli used in the Simon tasks for the Box and No Box conditions. The fixation point was a plus sign in the center of the screen. Stimuli (gray triangles), appeared alone or within a box frame to the left (L) or right(R) of fixation in and inner (I) or outer (O) positions relative to the fixation point.

In the second version (Box), the identical procedures were used, but a square box frame surrounded the visual field for the inner and outer positions for triangle appearance in each visual hemi field. Thus, the stimulus was aligned with the left or right side of the box, separated from the edge by 3mm. The second version also included the 20 practice trials. All participants were tested in both versions, with order of presentation counterbalanced within sex. The congruent and incongruent stimuli for both versions of the Simon tasks are depicted in Fig 1.

### Data analyses

An individual’s mean reaction time, excluding individual trials where reaction times exceeded 2000ms, was used for analysis; Data from one female participant was discarded due to variability in RT that exceeded 3 standard deviations from the female average, leaving 53 females in the analysis. Simple reaction times were calculated using the mean of the last 10 trials of the 20 practice trials and analyzed in a 2 (sex) X 2 (order) X Left/Right side of fixation ANOVA. For the experimental conditions mean RT and accuracy were analyzed using a 2 (Sex) X 2 (Order of presentation) X 2 (Condition: Box, No Box) x 2 (Right/left side of fixation) X 2 (Congruency; congruent, incongruent) X Position (Inner vs Outer) ANOVA with repeated measures over the last 4 factors. A Greenhouse-Geisser correction was applied if the sphericity assumption was violated. Bonferroni corrections were used for post-hoc comparisons.

## Results and discussion

### Reaction time

Simple reaction time was 315ms for males and 323 for females (p = 0.07). Across the two variations of the Simon tasks, the overall choice reaction time was significantly longer in females compared with males (F[1,89] = 26.27; p<0.0001; 542 vs 450ms; Cohen’s d = 1.75).

**Fig 2:**
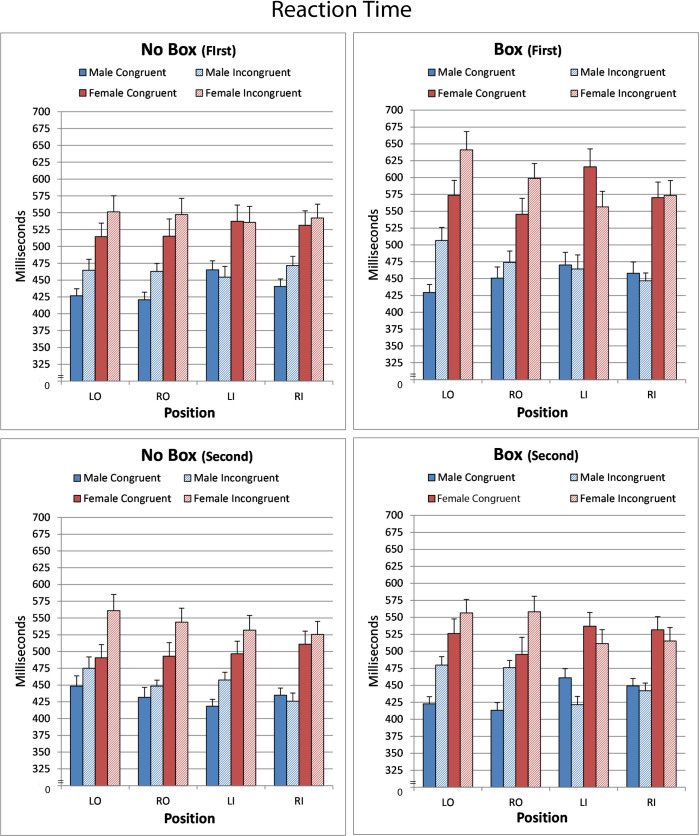
Depicted are reaction times (mean ± sem) for congruent and incongruent stimuli across condition, order of presentation and position. L = left; R = right; O = outer; I = inner. See results for statistical significance.

Overall choice reaction times were significantly faster in the No Box compared to the Box condition (F[1,89] = 16.34; p = 0.0001; 486 vs 505). However, this difference was influenced by order of presentation (BoxNoBox X Order; F[1,89] = 26.98; p<0.0001) and sex (BoxNoBox X Order x Sex; F[1,89] = 6.11; p = 0.015). As shown in Fig 2, the impact of the frame in the Box condition was greater in females when the Box condition was the first condition tested. The female increase in reaction time in the Box condition compared to the No Box condition was three fold greater than males (65 vs 19ms). In the opposite order of testing, however, the difference between the two conditions was similar in both sexes and slightly faster in the No Box condition (–5.4 vs –5.2), indicating that experience gained from the initial testing of the Simon task without the frame induced a similar degree of attentional suppression to this distracting information in males and females.

Reaction time to identify stimuli presented on the left side of fixation was 10 milliseconds longer compared with the right (F[1,89] = 5.40; p = 0.022). This main effect interacted with order (F1,89] = 6.09; p = 0.015, reflecting a significantly longer overall average reaction time of 13 milliseconds when the Box condition was the first presented compared to the reverse order.

### Simon effect

As anticipated, the tasks showed a strong positive Simon effect, the incongruent stimuli requiring longer time to process than congruent stimuli (F[1,89] = 49.22; p<0.0001) by an average of 12 milliseconds. A separate analysis of covariance using overall reaction time and the Simon effect revealed no significant association with sex.

**Fig 3:**
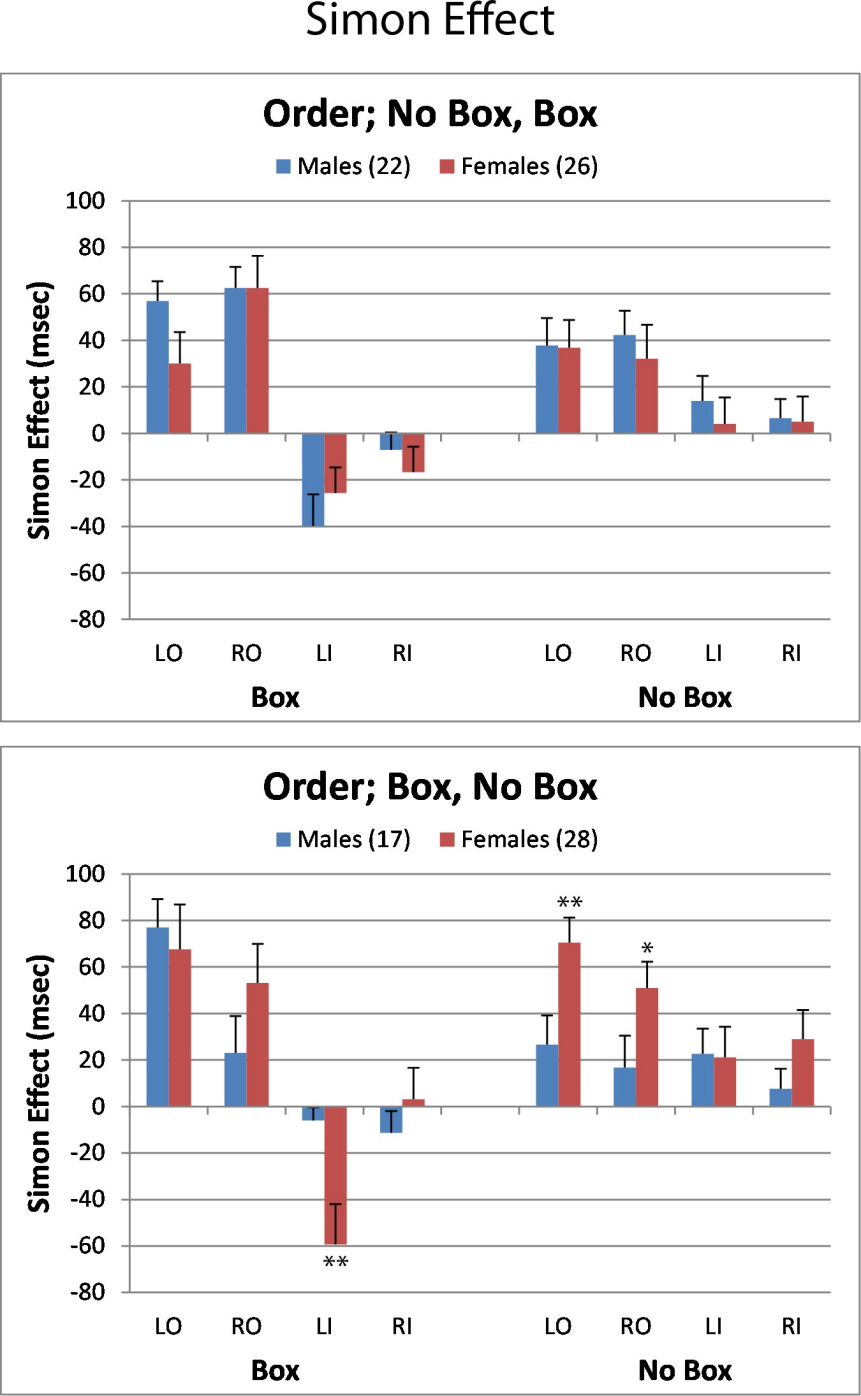
Simon effect is depicted as the mean difference in RT for the incongruent minus the congruent stimulus. Post hoc sex differences for individual conditions are identified by *(p<0.05) or **(p<0.01).

Significant main effects were found for Box/NoBox (F[1,89] = 4.05; p = 0.047 and Position (F[1,89] = 113.5; p<0.0001). Interactions included Order X Position X Left/Right (F[1,89] = 4.22; p = 0.042, Position X Left/Right (F[1,89] = 5.10; p = 0.026, Position X Sex X Order (F[1,89] = 3.92; p = 0.050, and Box/NoBox X Position (F[1,89] = 35.15; p<0.0001). Subsequent analyses of the Simon effect were conducted for each presentation order, the results of which are depicted in Fig 3.

### Order of presentation 1: NoBox, Box

In this order, the simple version of the Simon task was presented first. The analysis identified a main effect for Position (F[1,46] = 90.47; p<0.0001), which was reflected in the smaller Simon effect in the inner positions compared to the outer. This effect interacted with the BoxNoBox condition, where the direction of the Simon effects observed in the No Box condition, which was tested first, were enhanced by the presence of the Box (F[1,46] = 24.07; p<0.0001). As shown in Fig 3 (top panel), the presence of the Box frame enhanced the positive Simon effect in the outer position in the No Box condition and created a significant negative Simon effect in the inner positions. Thus, the presence of the frame increased the time needed to resolve the conflict in the outer position between the position of the stimulus and the hand response, but eliminated the conflict for inner position and made it easier to detect than congruent stimulus. In addition a BoxNoBox X Position interaction (F[1,46] = 24.07; p<0.0001) showed that the Simon effect was significantly greater in the outer positions of the Box condition compared to the No Box condition.

The analysis also revealed an interaction for Box/NoBox X Position X Left/Right X Sex (F[1,46] = 7.63; p = 0.008. This is reflected in the greater difference in the left inner position between the Box and No Box conditions in males compared to females. Within each condition for this order of presentation, separate analyses detected no sex effects in the Simon effects.

### Order of presentation 2: Box, NoBox

In this order, the response to the Box environment was significantly different from participants who had prior experience with the No Box condition, with sex differences found that were not observed in the reverse order of presentation. The analysis yielded a main effect for Position (F[1,43] = 37.60; p<0.0001), as well as interactions for Position X Left/Right (F[1,43] = 8.47; p = 0.006), BoxNoBox X Position (F[1,43] = 14.07; p = 0.0005), Position X Left/Right (F[1,43] = 9.83; p<0.0031), and a BoxNoBox X Position X Left/Right X Sex interaction (F[1,43] = 7.63; p = 0.008).

As shown in the bottom panel of Fig 3, in the outer positions of the Box condition, there were large positive Simon effects, but reverse Simon effects for the inner positions. This pattern was similar to that found in the Box condition when it was the second condition presented, but selectively exaggerated. The main difference is the female response in the left inner position shown in the bottom panel, where their reverse Simon effect was nearly 10 fold greater than males (–59 vs –6ms; p<0.01; d = 0.81). In addition, there was a sex difference in the Simon effects observed in the No Box condition in the outer positions, indicating a carryover influence of the initial experience with the frame in the Box condition. The positive Simon effects in females were more than twice that of males (**LO**: 70 vs 27ms; p<0.01; d = 0.77, **RO**: 51 vs 17ms; p<0.03; d = 0.65).

### Accuracy

**Fig 4:**
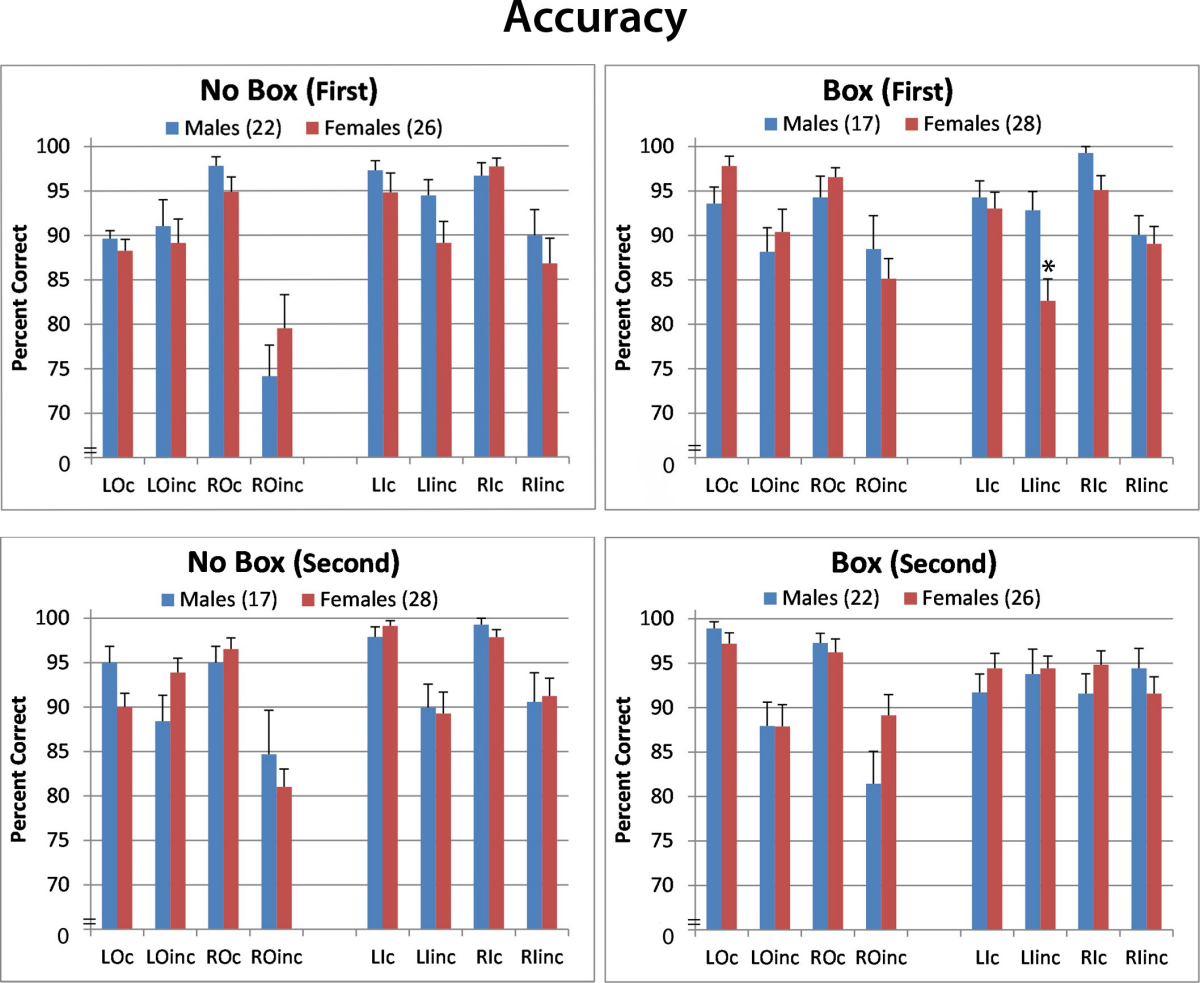
Accuracy (mean ± sem) for congruent and incongruent stimuli in males and females across Box/No Box conditions, order of presentation, Left/Right side of presentation, and position. *p<0.05 from opposite sex. L = left; R = right; O = outer; I = inner; c = congruent; inc = incongruent.

Overall accuracy was higher for congruent versus incongruent stimuli (F[1,89] = 41.46; p<0.0001; 95.4% v 88.4%). Overall accuracy was highest in the No Box condition when it was the first presented (BoxNoBox X Order (F[1,89] = 7.72; p = 0.007). Accuracy was lowest in the right outer position (F[1,89] = 10.45; p = 0.002). The only sex effect observed was in the left inner position of the Box condition when it was the first presented (Position X Congruency X Left/Right X Sex; F[1,43 = 7.32; p = 0.009). As shown in Fig 4, female accuracy for incongruent stimuli presented in the left inner position was significantly lower than males (93% vs 85%; d = 0.45). This is the same position where females showed a large negative Simon effect, meaning that they were significantly faster to identify the incongruent stimulus than the congruent stimulus.

## General discussion

Evidence supporting a sex-related bias in processing object/location relationships was revealed in the order in which the two versions were tested. No sex difference in the Simon effect was found when the No Frame version was the first presented, nor was there a sex difference found in the Frame condition that followed. Because the No Frame condition was the participant’s first experience with the tasks, the performance requirements were established without the presence of the irrelevant Frame that was present in the second version. The absence of a sex difference in the Simon effect with this order shows that the initial encoding of task requirements was similar in males and females, and both subsequently adapted to the presence of the frame in the same manner.

The hypothesized sex bias emerged when the more complex Frame version was the first tested. Since this was the participant’s first experience of the Simon task, the presence of the frame was now incorporated as part of the task environment. Females showed a large reverse Simon effect in the left inner position compared to males (-59 vs -6 msec), indicating that the additional information proximal to the stimulus helped them to more efficiently respond to incongruent stimulus. In this position, the congruent stimulus points left, with the back line of the triangle forming a parallel relationship with adjoining wall of the frame. The incongruent stimulus points to the right, where it adjoins the vertical wall of the frame to form a perpendicular relationship. The sex difference in the reverse Simon effect is similar to that observed by Simon et al. [9], where a large reverse Simon effect was observed in females, but not males.

When the frame was part of the initial experience with the test environment, it influenced the Simon effect in a sex-related manner in the No Frame version. While both sexes exhibited positive Simon effects in the outer positions, the female response was twice the size of males. Thus, it appears that the experience of the frame as part of the first test was implicitly coded more strongly in females, leading to a greater interference when the frame was absent.

The lack of a sex difference in the Simon effect when the No Frame condition was the first presented is consistent with previous studies that used relatively simple versions of the Simon task [2, 8–9,12], but stands in contrast with two more recent studies showing a larger positive Simon effect in females [10, 11]. The difference in those results from what we observed may be related to a procedural difference, wherein online feedback was provided to participants when they made an error regarding congruency. The feedback induced a small additional delay between trials and slowed the reaction time on the next trial in both males and females. However, the impact on reaction time in females was twice that of males. Stoet [11] removed the trials following feedback from the analysis and still found a larger sex difference in the female Simon effect. However, removal of this trial cannot rule out a lingering effect due to a stronger reaction in females to negative feedback. This possibility is supported by studies showing greater physiological sensitivity to negative feedback in females and its interference with task performance [36–38].

Accuracy in the Simon tasks was similar in men and women were similar, with the exception of the left inner position of the Frame version when it was the first presented. In this case, female identification of the incongruent stimulus was significantly poorer compared to males. This is the same position where females showed the large reverse Simon effect. The reason for the positional specificity of the sex difference is unclear, but suggests a role for sex differences in hemispheric organization [39]. It’s of interest that Efron et al. [40] found a similar sex-related positional effect in a discrimination study involving visual pattern recognition. Eighty-two participants were required to detect a target pattern that appeared for 133 msec on one of 6 boxes positioned horizontally to the right and left of fixation (3 on each side). The error rates for females in the inner two boxes on the left side of fixation were more than double that of the forty one males. No sex difference was found for the other positions. However, it should be noted that the Efron study used a stimulus duration designed to eliminate saccadic eye movements, which was not the case for the 400 msec duration used in the present study. The authors make a case for the sex difference arising from a stronger tendency in females to employ a left to right visual scanning strategy. This is supported by the work of Adam et al. [41], which identified a serial left to right strategy for females in a 4 choice discrimination tasks, but dichotomizing, split-half strategy for males.

Finally, although the overall reaction time of females was 10–15% longer than males to process both congruent and incongruent stimuli, this large difference was not related to the Simon effect. The large sex difference in overall reaction time suggests differential processing in the integration of object characteristics. Similar differences in reaction time, without differences in accuracy, have been observed in other types of choice discrimination of basic visual elements [16–17, 42]. This sex difference is not a general aspect of discrimination studies, since women are faster than men at making a local discrimination in Navon type tasks and show faster processing speed for non-spatial tasks measuring intellectual performance [43–49]

The relevance of the current findings to a broader understanding of cognitive sex differences is the focus it places on early processes underlying the perception of objects within the context of their environment. We know that biological, social and cultural interactions contribute to sex differences in neural connectivity [48], but understanding how these factors interact to create sex differences in specific types of tasks has proven to be elusive. From a functional perspective, an underlying neural difference in cognitive strategy has been widely accepted for decades [49–51]. The operational level involved to explain such differences has generally focused on top-down processes and varies in accordance with the higher cognitive tasks employed. These fall broadly into categories related to selective attention, verbal abstraction, and space relations, with supporting evidence for sex differences often provided by neural activation patterns during task performance and functional brain organization [52–54]. However, with a better understanding of how development of the dorsal and ventral streams contributes to higher cognition, there is increased interest in how sex differences in lower level processing within these streams can serve as filters for attention and thereby build a foundation for the emergence of sex differences in higher level tasks [55,56]. Support for this perspective arises from studies of infants and children showing sex-related biases for attending to objects versus location [57], suggesting that these early biases in processing of sensory information and its integration into associational networks are strongly inherent. The present study shows similar biases are still present in adults, supporting a link between implicit coding of allocentric relationships between objects and location and sex differences in higher cognitive skills that rely on processing of these elements.

## Supporting information

S1 File This is the experimental data excel file for analyses. (XLSX)
